# Mapping of the biomedical literature evaluation competencies based on pharmacy students’ feedback

**DOI:** 10.1186/s12909-016-0583-7

**Published:** 2016-02-12

**Authors:** Soumana C. Nasser, Aline Hanna Saad, Lamis R. Karaoui

**Affiliations:** Department of Pharmacy Practice, School of Pharmacy, Lebanese American University, P.O. Box: 36 (S23), Byblos, Lebanon

**Keywords:** Assessment, Student mapping, Curriculum mapping, Biomedical literature evaluation, Learned curriculum

## Abstract

**Background:**

This study aims to map the learned curriculum based on students’ feedback regarding the biomedical literature evaluation competencies in a pharmacy curriculum, to evaluate teaching methods and to report students’ longitudinal self-assessment of their achievement of related learning outcomes as they progress from didactic to experiential courses.

**Methods:**

The biomedical literature evaluation competencies were mapped in three courses delivered during different pharmacy professional years (PPY): Drug Information and Literature Evaluation (PHA421) offered in the second PPY, Pharmacoeconomics (PHA557) and Professional Pharmacy Practice Experience-Hospital/Drug Information Services (PHA570) offered in the third PPY. A unified survey was developed to collect information from students at the beginning and completion of these courses. Survey results were then compared to school assessment data of identified courses for triangulation of findings.

**Results:**

Listed student learning outcomes are consistently achieved through all three courses with more assertion from the students at the completion of the applied experiential course PHA 570 (>90 % agree or strongly agree). In terms of delivery methods, 84 % of students perceived the benefits of active learning methods in reinforcing acquired skills and increasing confidence in knowledge and critical thinking in a less stressful learning environment. Results shown at the end of each course indicate a favorable student response from one course to the next where almost all students replied with ‘agree to strongly agree’ to survey questions assessing their readiness to critically evaluating trials (72 %, 96 % and 92 %) in PHA421, PHA557 and PHA570, respectively. Study findings are in congruence with school assessment database of the selected courses.

**Conclusion:**

Formative assessment results demonstrated acquisition of required analytical skills, and completion of course learning outcomes as students progressed from introductory to advanced courses covering the biomedical literature component.

## Background

There is growing interest in global pharmacy education to create a learning environment where students take ownership of their learning, shift from rote memorization to critical thinking, and become motivated, confident and active life-long learners [1]. In congruence with these goals, the 2013 report from the Center for the Advancement of Pharmaceutical Education (CAPE) delineates four broad domains of pharmacy education: foundational knowledge, essentials for practice and care, approach to practice and care, and personal and professional development. The corresponding subdomains envision the graduating pharmacist as a problem solver, communicator, collaborator, learner and educator. He/she is a learner with the ability to critically evaluate scientific literature related to drugs and diseases to enhance clinical decision making [2].

Accordingly, the Lebanese American University School of Pharmacy (LAU SOP), which offers the only Accreditation Council for Pharmacy Education- (ACPE)-accredited doctor of pharmacy (Pharm.D.) program outside the United States, pledges to equip graduates with the necessary skill set to deliver quality patient-centered care. Its curricular philosophy binds the program to the use and integration of teaching and active learning methods throughout the didactic and experiential curriculum to maximize students’ problem solving skills and the pursuit of lifelong learning. Previous studies demonstrated a positive impact of weekly active learning activities in a Drug Information and Literature Evaluation course associated with increased student ability and confidence in all course objectives [3]. Similarly, cooperative learning using the jigsaw method in a drug assignment activity was successful in improving the students’ critical thinking skills [4].

As LAU SOP students matriculate through the program and transition from Introductory Pharmacy Practice Experiences (IPPEs) to Advanced Pharmacy Practice Experiences (APPEs) their learning process is geared towards a more independent and active approach and a better understanding of the biomedical literature. In fact, the content and delivery methods employed in multiple courses across the pharmacy curriculum were designed to prepare students to evaluate the biomedical literature through the implementation of active learning methods.

The School of Pharmacy Curriculum Committee and faculty undertook the endeavor of mapping the entire LAU SOP curriculum to better align the intended, enacted, assessed and learned curricula. The intended curriculum indicates what the students are expected to learn or faculty are expected to deliver, while the enacted curriculum describes the actual content that is taught or delivered, in the classroom based on faculty feedback. The learned curriculum refers to the knowledge and skills acquired by students during the schooling process. The assessed curriculum represents a system of processes and tools that are used to determine the extent to which students are acquiring or have acquired knowledge and skills [5]. Previous research demonstrated the positive value of curriculum mapping in program assessment and evaluation [6]. Curriculum mapping considers when, how (teaching method) and what is taught, in addition to the assessment measures used to demonstrate the achievement of expected student learning outcomes [7]. It is a continuous quality assurance tool that helps faculty understand course content, delivery, depth and breadth of covered competencies. The ensuing map helps quantify and spatially and visually represent selected curricular elements such as the extent to which content is delivered in each professional pharmacy year. It provides a platform for curricular analysis and subsequent generation of improvement plans [8, 9]. When referring to mapping, it is usually the one completed by faculty for the taught/enacted curriculum. Students’ mapping of the learned curriculum is a rare activity that is seldom documented in the literature. The results of a study designed to examine whether the intended outcomes in a teacher education course could be identified accurately by the students, showed that “the outcomes identified by students were easily classified into the intended thematic curriculum of the course” [10]. Another study reported the student mapping endeavor of 89 different courses in a public health curriculum using factor analysis and observed that student opinion can provide valuable and unbiased information about courses [11]. To our knowledge, the formative assessment of pharmacy students’ perception of the biomedical literature evaluation at different levels of learning has not been undertaken in the Middle Eastern context nor fully reported in the literature. The primary objective of this study is to map the learned curriculum based on students’ feedback pertaining to the biomedical literature evaluation competencies of the LAU SOP curriculum and its related teaching methods. The secondary objective is to report the students’ longitudinal self-assessment and formative assessment of their achievement of this learning outcome as they advance from didactic to experiential courses.

## Methods

### Course learning outcomes and curriculum mapping

The biomedical literature evaluation competencies of the pharmacy curriculum were mapped in three courses delivered across different pharmacy professional years (PPY): Drug Information and Literature Evaluation (PHA421) offered in the second PPY, Pharmacoeconomics (PHA557) and Professional Pharmacy Practice Experience- Hospital/Drug Information Services (PHA570) offered in the third PPY. According to the curriculum philosophy, a major purpose of these courses is to prepare students to evaluate the medical literature sequentially and at the appropriate depth and breadth, transitioning from introductory level to reinforced and then applied levels. In the Drug Information and Literature Evaluation course (PHA421), the biomedical literature evaluation skills are introduced for the first time in the pharmacy curriculum. Course learning outcomes include (1) differentiating (Bloom’s taxonomy [12] – analysis category) between study designs and study characteristics; (2) identifying (Bloom’s taxonomy – knowledge category) study power, bias, and confounders; (3) interpreting (Bloom’s taxonomy – evaluation category) study results; and (4) determining (Bloom’s taxonomy – evaluation category) internal and external validities of a study.

In the Pharmacoeconomics course (PHA 557), students are introduced to the concept of Pharmacoeconomics (PE) and trained to evaluate the pertaining literature while building on their acquired knowledge from PHA421. Evaluating the literature is reinforced in this course through analyzing pharmacoeconomics studies including (1) differentiating (Bloom’s taxonomy – analysis category) PE models, (2) identifying (Bloom’s taxonomy – knowledge category) study characteristics (type of models, perspectives, type of costs, data recourses), (3) determining (Bloom’s taxonomy - evaluation category) study validity, and (4) generating (Bloom’s taxonomy- synthesis category) recommendation to facilitate decision-making.

The same learning outcomes of PHA 421 and 557 are then covered at an applied level in the Professional Pharmacy Practice Experience (PHA 570), whereby students are trained to apply and conduct literature evaluation of several articles that encompass drug information consults on specific topics, drug information queries, and newsletters. In addition, the literature evaluation activities incorporated appraisal of diverse study designs ranging from retrospective to randomized clinical trials, meta-analysis as well as systematic reviews. Through this hands-on experience, students apply their biomedical literature evaluation skills while interacting with other health care providers to ensure optimal evidence-based pharmacotherapy and patient care delivery.

As noted, the curriculum is designed so that the same learning outcomes were covered in all three courses at different depth and breadth: introduce in PHA 421, reinforce in PHA 557 and apply in PHA 570.

### Active-learning methods

The approach used in implementing active learning as a teaching method in these three courses is based on strategies described by Gleason et al. that support the use of active-learning in didactic and experiential courses in pharmacy curricula [13]. The teaching methods implemented at varying depth in all three courses mostly conform with the strategies described as ‘Minutes Writes’, ‘Student Presentations’, ‘Audience Response Systems’, and ‘Case Studies’.

Through the “Minutes Writes”, students are often asked an open-ended question regarding evaluation of a scientific article and given a few minutes to prepare a response. In ‘Student Presentations’, students are asked to work in groups on an assigned drug related question requiring literature evaluation. They present their work during the last two weeks of the semester where they discuss their topic with students from other groups. In ‘Audience Response Systems’, assimilation of course concepts is assessed during lecture time, by posing questions, engaging the entire class through passive or active participation to demonstrate their understanding of or confusion about the course materials. ‘Case studies’ are also used allowing students to work in groups or individually in solving drug information consults and queries while applying their knowledge and skills in literature search and evaluation.

### Survey questionnaire

A unified survey questionnaire was developed by the course instructor to collect information from students in the three identified courses. The survey was completed at the beginning and at the completion of each of these three courses with answers provided anonymously. The survey was validated prior to its use as it was an online institutional survey run for ACPE accreditation purposes from the assessment database. The estimated time for completing the survey was 5–10 min for each course, and adopted the five-point Likert scale. It aimed at assessing students’ perception regarding: (1) course learning outcomes covering literature evaluation competencies; (2) the effectiveness of implementing active learning activities as delivery method in these courses; and (3) longitudinal progression in acquiring literature evaluation skills throughout these three professional courses. The survey was conducted in the didactic courses PHA421 and PHA557, enrolling 68 and 72 students, respectively, and in one of the two sessions of the experiential course PHA570 enrolling 38 students during the academic year 2013–2014.

To validate and compare results, and in an effort to triangulate findings, these surveys were complemented with reliable school assessment data pertaining to these courses. Direct assessment of student learning outcomes was performed via embedded exam questions whereby students were asked to critically analyze the biomedical literature through several exam questions related to a full text trial and to different case scenarios retrieved from peer-reviewed journal articles. The exam questions required the students to address different aspects of biomedical literature evaluation from identifying the study design, characteristics, statistical tests, limitations and strengths, to further interpreting the statistical data and findings, and finally to determining the internal and external validities of the study. Indirect assessment was completed through student evaluations of courses and of student learning outcomes (SLOs) as derived from syllabi; such assessment helps in determining if all SLO listed in the course syllabi were covered by the instructor and the depth of coverage.

Statistical analyses were performed using SPSS version 18 software. Outcome measures were summarized using frequencies and percentages. *P* values below 0.05 were considered to be statistically significant. The association between categorical variables was evaluated using Pearson *χ*^2^ test or Fisher’s exact test where the expected cell count was less than 5. The Chi-square was used when comparing student’ responses on statements within courses and across courses and to report the *p*-values. This study was approved by the Lebanese American University Institutional Review Board and has been performed in accordance with the ethical standards as laid down in the 1964 Declaration of Helsinki and its later amendments.

## Results

Students’ mapping of the biomedical literature evaluation skills in PHA 421, 557 and 570 showed that the listed SLOs are consistently achieved through all three courses with more assertion from the students at the completion of the applied experiential course PHA 570 (>90 % agree or strongly agree). A response rate answering ‘agree to strongly agree’ of at least 80 % was obtained on learning outcomes addressing searching resources, analyzing statistical data, differentiating among study designs, and critically evaluating clinical or PE studies. Results in Table 1 highlight students’ responses indicating that the same learning objectives are sequentially delivered in these three courses at different depth. On average 84 % (range 78 % to 92 %) of students perceived that active learning methods facilitate students learning, improve progress, reinforce acquired skills, and increase confidence in knowledge and critical thinking in a less stressful learning environment. Results of Table 2 indicate a favorable student response rate when asked about delivery methods used in PHA 421, 557 and 570. Implementation of active learning in didactic courses revealed positive impact as team work learning activities was reported as a waste of class time by only 5 % and 6 % of students in PHA421 and PHA557 respectively. On the other hand, 48 % of students in the experiential course PHA570 found that team work on assignments and projects at the training site was a waste of time when compared to direct patient care that they could provide through this course. While 34 % of students in the introductory course (PHA421) found that the number of class hours was not enough to prepare them to adequately analyze clinical trials, this number decreased to 19 % in the reinforced didactic course (PHA557).

**Table 1:** Students’ mapping of biomedical literature evaluation skills-achieving similar learning outcomes in PHA 421, 557 and 570. *(percentages for answering either ‘agree’ or ‘strongly agree’)*

(At the end of each course, the student strongly agrees or agrees that the below SLOs are met)	PHA 421	PHA 557	PHA 570
	(*n* = 68) N (%)	(*n* = 72)	(*n* = 38)
Identify and conduct a literature search using search engines (PE^a^ database, secondary resources, PubMed, etc.)	56 (82 %)	63 (88 %)	34 (90 %)
Describe different study designs and characteristics (i.e. experimental vs. non experimental designs, different PE models, clinical trials)	57 (84 %)	58 (80 %)	37 (97 %)
Define and interpret statistical parameters (i.e. study validity and reliability, power, *p* values, type I and type II errors)	55 (81 %)	66 (92 %)	37 (97 %)
Critically evaluate clinical and/or PE studies (i.e. determine the study internal/external validities, apply PE in pharmaceutical care decisions making)	61 (90 %)	58 (80 %)	36 (95 %)

^a^PE: Pharmacoeconomics

**Table 2:** Students’ mapping of the biomedical literature evaluation skills – evaluating the delivery methods in PHA 421, 557 and 570. *(percentages for answering either ‘agree’ or ‘strongly agree’)*

	PHA421 (*n* = 68) N (%)	PHA557 (*n* = 72)	PHA570 (*n* = 38)
In-Class Team-Work Learning Activities:			
Number of class hours spend preparing me to critically analyze PE^a^/clinical trials were adequate	23 (34 %)	58 (81 %)	--
In-class learning activities (on assignments) facilitated my learning of class materials	54 (80 %)	57 (79 %)	--
In-class learning activities helped me improve my progress in analyzing PE/clinical trials	53 (78 %)	66 (92 %)	--
In-class discussing articles in a small group setting helped reinforce course materials more than studying alone	55 (81 %)	64 (89 %)	--
In-class discussing articles increased my confidence in my knowledge for evaluating PE/clinical trials	59 (86 %)	66 (92 %)	--
Outside Class in Addition to In-Class Team-Work Learning Activities:			
In/out-side class team-work on assignments/article analysis increased my confidence in knowing how to analyze clinical/PE articles	45 (66 %)	61 (85 %)	34 (89 %)
In/out-side class team-work discussing/presenting article as presentation helped reinforce my ability to evaluate articles	49 (72 %)	61 (85 %)	34 (89 %)
In/out-side class team-work applying course materials on projects/assignments provided a less stressful learning environment	49 (72 %)	67 (93 %)	31 (83 %)
In/out-side class learning activities on assignments ‘in didactic courses’/working in the Library ‘in the experiential course’ were a waste of class time	3 (5 %)	4 (6 %)	18 (48 %)

^a^PE: Pharmacoeconomics

Study objective 2 focusing on students’ longitudinal self-assessment of their readiness to evaluate the biomedical literature was assessed prior to and at the end of each course and reported in Table 3. Students’ preparedness to evaluate biomedical literature was shown to improve within courses (before and after taking each course, all *p*-values < 0.001), and across courses (from the introductory level course to the applied level course, *p*-value = 0.003 for the question on external validity). Prior to the beginning of didactic courses (PHA421 and PHA557), very few students were ready for the learning outcomes of these introductory-reinforced level courses, such as knowing how to evaluate different types of clinical or PE studies (8 % and 9 % in PHA421, 11 % and 7 % in PHA557). On the other hand and as expected, prior to the beginning of the experiential course (PHA570), a significantly higher percentage of students already knew the learning outcomes of this applied level course (67 % and 42 % in PHA570). Results shown at the end of each course indicate statistically significant improvement from one course to the next where almost all students replied with ‘agree to strongly agree’ to survey questions assessing their readiness to critically evaluating trials (72 %, 96 % and 92 %), and determining external validity (65 %, 85 % and 89 %) in PHA421, PHA557 and PHA570, respectively. Table 4 summarizes a comparison between ‘Direct Assessment’ and ‘Indirect Assessment’ of specific learning outcomes covered through the three PHA courses and addressed in this study based on school assessment findings. Direct association was shown between students’ learning satisfaction of covered course materials (indirect assessment completed during the last class session) and acquired knowledge demonstrated in the exam results (direct assessment performed during the final exam). The high ranking observed in the indirect assessment of specific learning outcomes done through student’s feedback survey was confirmed by the observed similar high ranking of the same learning outcomes included in the direct assessment done through embedded questions in the midterm and final exams. Most importantly, results indicated that the final learning outcome ‘critical article evaluation’- which was the culmination of all other learning outcomes in each of these three courses - was achieved in 92 % and 87 % of students upon their completion of PHA421 in PPY2 and PHA570 in PPY3, respectively.

**Table 3:** Students’ longitudinal self-assessment of their readiness to evaluate the biomedical literature: clinical trials in PHA421/PHA570, and pharmacoeconomic (PE) trials in PHA557: *(percentages for answering either ‘agree’ or ‘strongly agree’)*

	PHA421 (*n* = 68) N (%)	PHA557 (*n* = 72)	PHA570 (*n* = 38)	*p-*value (across courses)
Prior to the BEGINNING of this course:				
I already knew how to differentiate among different types of clinical/PE^a^ study designs.	5 (8 %)	8 (11 %)	25 (67 %)	<0.001
I already knew how to evaluate clinical/PE trials	6 (9 %)	5 (7 %)	16 (42 %)	<0.001
By the END of this course:				
I know how to determine the external validity of a PE/clinical trial	44 (65 %)	61 (85 %)	34 (89 %)	0.003
My perception on critically evaluating a PE/clinical trial significantly improved	49 (72 %)	69 (96 %)	35 (92 %)	<0.001
*p-*value (value within the same course, from beginning to end of each course)	<0.001	<0.001	<0.001	

^a^PE: Pharmacoeconomics

**Table 4:** Direct and indirect assessment of certain course specific learning outcomes related to biomedical literature evaluation covered in the final exam of each course

	Direct assessment	Indirect assessment
PHA421, *N* = 68	N (%)	N (%)
Evaluate clinical trials published in the medical and pharmaceutical literature	63 (92 %)	61 (90 %)
Describe different study designs including experimental and non-experimental designs.	60 (88 %)	57 (84 %)
Interpret study statistical analysis including *p*-values, and type I and II errors	55 (81 %)	55 (81 %)
PHA557, *N* = 72		
Critically evaluate a pharmacoeconomic article (i.e. apply PE^a^ in pharmaceutical care decisions making)	53 (74 %)	58 (80 %)
Define type I error, Type II error, power, effect size, *p*-value, and the relation among them	66 (92 %)	66 (92 %)
PHA570, *N* = 38		
Critically evaluate clinical trials (e.g. identify study characteristics, interpret the statistical data, and determine the study internal and external validities)	33 (87 %)	37 (97 %)

^a^PE: Pharmacoeconomics

## Discussion

This study reports on students’ mapping of the learned curriculum by providing their feedback on the achievement of the biomedical literature evaluation competencies through three different courses in two professional pharmacy years. Both content and delivery methods were evaluated by the students and perceived to be achieved. LAU SOP faculty recently completed a similar endeavor whereby they mapped the taught/enacted research component of the curriculum against the CAPE 2004 educational outcomes and Appendix B of the ACPE guidelines [14]. Comparing the mapping of the enacted curriculum to that of the learned curriculum confirmed that both faculty and students agree that the biomedical literature evaluation skills are well integrated in these three courses (SLOs achieved from faculty and students perspectives), progressively (same SLOs covered in three courses in two different PPY) and at the appropriate depth (introduced, reinforced and then applied). An ACPE required element of program-level assessment, mapping is a reliable mechanism to show concordance between the different components of the curriculum. Curricula maps help in visualizing the link between competencies-based assessment and program-level outcomes [8]. Students’ comprehensive mapping of the entire curriculum might be challenging as they lack the ability to judge cumulative curricular coverage along with the appropriateness of depth and breadth of delivery [6, 7, 10, 11]. However, when focused on specific skill sets in selective courses as completed in this study, students were successful in assessing the content coverage as confirmed by the direct and indirect assessment methods (Table 4).

The available literature details varied students’ reaction to the implementation of active learning methods from acceptance to a process needing time for integration [15, 16]. These findings concur with our results as student responses on delivery methods used in introductory and applied courses were favorable. Our findings converge with those of Kai et al. whereby third year pharmacy students were asked to present their feedback on active learning method implemented in a pharmacotherapy course [17]. Results have shown that the majority of students (71 %) agreed or strongly agreed that their ability to learn the material presented was enhanced. Students also reported that their motivation was improved while their anxiety was diminished with active learning [17]. By engaging students in the learning process, they develop critical thinking, problem-solving and cognitive skills needed to apply the knowledge they gain [13, 18]. Students’ intellectual curiosity and critical judgment developed through active learning are crucial to make informed, rational and evidence-based pharmacotherapeutic decisions. Active learning is a teaching approach that requires student participation in classroom or experiential activities that are well-designed by teachers [13]. It fosters a two-way communication to enhance student comprehension, stimulate interest, and emphasize on attitudes and values to promote student ability and confidence in achieving course learning outcomes [3, 13].

Students’ self-assessment for their readiness to evaluate the biomedical literature is one of the lifelong self-learning pillars. Once these literature evaluation skills are achieved, students will be able to utilize them continuously to critically think through drug information questions while caring for patients at the bedside and other health care settings.

The limitations of this study include not performing correlations of the study outcomes on biomedical literature with the class averages for the student groups that completed the survey. Another limitation to this study is the fact that different student cohorts completed the survey for the three different courses. It would have been ideal if the same student cohort was followed as they progressed from one course to another. While mapping this component of the curriculum, students did not provide ideas for improvement but only assessed current patterns. Their active input on areas needing developments would have added significantly to this initiative. Finally, as feedback was not consistently provided to students at end of each session, student’ self-regulated learning, a building block of active learning and formative assessment, was not evaluated in the survey questionnaire.

## Conclusion

Biomedical literature evaluation skills are necessary assets to pharmacists who are the drug information experts. Students’ mapping of the curriculum content and delivery methods of the biomedical literature evaluation skills is an assessment tool of these skills in the curriculum. In this study, students’ mapping of biomedical literature student learning outcomes validated other assessment methods employed by the program and triangulated well with faculty feedback.

## Abbreviations

CAPE: Center for the Advancement of Pharmaceutical Education
ACPE: Accreditation Council for Pharmacy Education
LAU SOP: Lebanese American University School of Pharmacy
PharmD: Doctor of pharmacy
IPPE: Introductory Pharmacy Practice Experiences
APPE: Advanced Pharmacy Practice Experiences
PPY: professional pharmacy year
